# Pectin obtention from agroindustrial wastes of *Malus domestica* using green solvents (citric acid and natural deep eutectic solvents). Chemical, thermal, and rheological characterization

**DOI:** 10.3389/fchem.2024.1504582

**Published:** 2025-01-06

**Authors:** Carolina Gómez Vargas, Nora Marta Andrea Ponce, Carlos A. Stortz, Eliana Noemi Fissore, Pablo Bonelli, Carlos Mauricio Otálora González, Lía Noemí Gerschenson

**Affiliations:** ^1^ Departamento de Industrias, Facultad de Ciencias Exactas y Naturales, Universidad de Buenos Aires, Buenos Aires, Argentina; ^2^ Instituto de Tecnología de Alimentos y Procesos Químicos (ITAPROQ), CONICET - Universidad de Buenos Aires, Buenos Aires, Argentina; ^3^ Departamento de Química Orgánica-CIHIDECAR, Facultad de Ciencias Exactas y Naturales, Universidad de Buenos Aires, Ciudad Universitaria, Buenos Aires, Argentina

**Keywords:** pectin production, apple wastes, citric acid, NADES, ultrasound, characterization and functionality

## Abstract

The use of green solvents, citric acid (CA), and natural deep eutectic solvents (NADES) for the obtention of pectin from wastes (pulp and peel) of *Malus domestica* was studied. The NADES used comprised citric acid–glucose–water (N1) or lactic acid–glucose–water (N2). The fractions rich in pectin obtained after exposure to NADES showed lower yield (≈4 g/100 g CA vs. ≈ 11 g/100 g CA), equal to or lower degree of methoxylation (53–71 mol/100 mol CA vs. 73 mol/100 mol CA), equal to or greater content of uronic acid (50–63 g/100 g CA vs. 51 g/100 g CA) than those isolated with CA, and the ones obtained from peel were the most thermally stable. These pectins showed greater linearity, shorter branch lengths, and lower arabinose content than those obtained with CA. The neutral sugars present in the highest concentration in all the isolated fractions were arabinose, xylose, galactose, and rhamnose. Glucose was also detected, probably due to contamination with starch. Their aqueous solutions showed pseudoplastic behavior. The effect of ultrasound assistance was preliminarily evaluated in the production of pectic fractions using N2, observing higher yields (13–18 g/100 g), in general, a higher concentration of uronic acid and a higher degree of methoxylation when compared with the extraction without US. They also showed greater arabinose content (less degradative treatment), lower glucose content (increased purity), and higher rhamnogalacturonan I (RG-I) content. It is known that RG-I is linked to pectin bioactivity and rheological behavior. The green solvent techniques assayed allowed obtaining fractions rich in uronic acid with different chemical, thermochemical, and rheological characteristics. In the case of isolation with NADES, the yield was low, but preliminary tests with ultrasound assistance showed that it is possible to overcome this limitation.

## 1 Introduction

The high level of food production generates a large amount of agroindustrial waste with diverse characteristics. The United Nations (FAO, 2012) estimates that approximately one-third of all food produced in the world is lost or wasted, which is equivalent to approximately 1.3 billion tons per year. Fruits, vegetables, roots, and tubers account for approximately 40%–50% of the total food waste (Ravindran et al., 2018).

According to the Food and Agriculture Organization of the United Nations (FAO), global apple production in 2022 was approximately 95 million tons (Food and Agriculture Organization of the United Nations, 2024). The industrialization of this fruit produces between 25% and 30% of waste called apple pomace (Antonic et al., 2020).

Pectins are complex macromolecules constituted of homogalacturonan (HG), rhamnogalacturonan I (RG-I), and rhamnogalacturonan II (RG-II), linked by covalent unions through their backbones. More than half (65%) of the pectins comprise HG, that is, galacturonan blocks in which the anhydrous D-galacturonic acid (GalA) is connected through 1,4-linked α-D-Gal*p*A residues. The RG-II is a galacturonan that constitutes 10% of the pectin molecule; it is a complex region in which galacturonan is highly substituted by 12 monosaccharides. RG-I presents a disaccharide repeat backbone of [4)-α-D-GalA-(1,2)-α-L-Rha-(1,] and, as such, it is not a rhamnogalacturonan; it constitutes ≈20–30% of the pectin macromolecule. Rhamnosyl residues are substituted by D-galactose [β-D-Gal*p*-(1 → 4)], giving origin to galactan chains and/or the oligomeric or polymeric side chains of arabinogalactan I, and/or short or large chains of α-1,5-linked L-arabinan, which can be branched with arabinan chains. These macromolecules can be classified into high (HM) and low (LM) methoxyl pectins. HM pectins have more than 50% of their carboxyl groups esterified with methanol, and LM pectins have between 5% and 50% of their carboxyls in this situation. This leads to diverse applications because the thickening and gelation conditions are different in each case (Gawkowska et al., 2018). Common sources of pectin are citrus peels and apple pomace (Gerschenson et al., 2021).

The most common technique for producing pectin from plant tissues is acid extraction. There are numerous studies about isolating pectin from apple, and, in general, they involve the use of strong acids such as hydrochloric or nitric acid (Canteri-Schemin et al., 2005) or organic acids such as citric or acetic (Luo et al., 2019). In other studies, this polysaccharide is obtained using enzymes (Wikiera et al., 2022).

Pectin extractions from plant tissues using environmentally friendly methods are currently considered the methods of choice for obtaining this polysaccharide. The considerations that guide the classification of methods as environmentally friendly are linked to the fact that they produce good yields and high-quality products in less time, using non-polluting solvents. The use of a smaller amount of solvent is also valuable because it saves resources. Environmentally friendly methods require the use of green solvents such as natural deep eutectic solvents (NADES), water, and supercritical fluids, as well as the assistance of green methods such as microwaves, ultrasound, and enzymes (Tien et al., 2022).

Ultrasound (US) is a process that involves the delivery of energy to a target with the objective of promoting the desired effect on the target. In the case of obtaining pectins from vegetable matrices, the application of US using a probe produces cavitation in the solvent due to the formation, growth, and collapse of bubbles (Chemat et al., 2017). This last phenomenon gives rise to the rupture of the biological membranes present in the tissues involved, facilitating the penetration of the solvent into the matrix and the release of the compounds of interest due to the increase in mass transfer. All of this reduces process time and generally increases performance (Gerschenson et al., 2021). On the other hand, the application of US can generate changes in the structure of the isolated pectins and their functionality. Dranca et al. (2020) studied obtaining pectin from apple pomace and observed that a conventional extraction with citric acid produced a higher pectin yield than an extraction with the same acid but assisted with ultrasound (23% vs. 9%). Likewise, ultrasound increased the galacturonic acid content and produced a lower methoxylation degree (DM) and an increase in the molecular mass of the isolated pectin. Idrovo Encalada et al. (2019) used carrot leftovers to evaluate the obtaining of pectin assisted by cellulase or hemicellulase. They observed that US pretreatment increased the extraction yield of LM pectins and decreased their DM and molecular mass but allowed obtaining calcium crosslinked gels, with higher elastic modulus than fractions isolated without US assistance. Wang et al. (2016) used hydrochloric acid to obtain pectin from grapefruit, isolating HM pectins. When the process was assisted with US, a polysaccharide with a lower molecular mass, lower DM, more branched chains, higher thermal stability, and lower apparent viscosity and elasticity was obtained.

NADES comprise a eutectic mixture of at least two natural compounds (i.e., amino acids, organic acids, sugars) in a specific molar ratio, giving rise to homogeneous mixtures. They are an emerging class of green solvents characterized by depressions in the melting temperature compared to those of the pure constituents (Hansen et al., 2021). They have a hydrogen bond donor and a hydrogen bond acceptor that determine their supramolecular structure, and also van der Waals interactions or even electrostatic forces can influence melting point depressions and other physicochemical properties of NADES (Santana-Mayor et al., 2021). Its advantages include abundant components that do not react with water and are biodegradable and non-toxic (Benvenutti et al., 2020).

According to our knowledge, there is scarce literature about

- The effect of different NADES on the extraction of pectins.
- The chemical structure, composition, and functionality of pectins isolated with NADES solvents and their differences in relation to those extracted with organic acids.
- The effect of assistance developed with different methods on extraction with NADES.
- The mode of use of NADES solvents for pectin extraction (i.e., full solvents or pretreatment followed by aqueous extraction).

Accordingly, the objective of the present investigation was the evaluation of the use of two selected NADES to obtain pectins from apple peel or pulp, comparing the polysaccharide characteristics with those obtained in the extraction developed with an aqueous solution of citric acid (pH 2.3). The characterization of the products obtained will allow us to evaluate the impact of the isolation method on the quality and functionality of the products and to conclude about the suitability of the proposed techniques.

## 2 Materials and methods

### 2.1 Materials

The apples (*Malus domestica*) Red Delicious variety were provided by Kleppe company (Cipolletti, Rio Negro, Argentina).

The chemicals used were lactic acid (85%–90%. Pro-Analysis, ACS), citric acid (Pro-Analysis, ACS), anhydrous D (+)-glucose (Pro-Analysis, ACS), and phenol crystals (Pro-Analysis, ACS) were provided by Cicarelli Laboratories (Santa Fe, Argentina). D- (+)-galacturonic acid monohydrate, Protein Standard BSA, and 3-phenylphenol were provided by Sigma Aldrich (Missouri, USA). Methanol ACS was provided by Panreac AppliChem, (Castellar del Valles, Spain). Ácido sulfúrico (98%. ACS) and acetyl acetone (≥99%) were provided by Anedra (Buenos Aires, Argentina). Folin Ciocalteu reagent was provided by Biopack (Buenos Aires, Argentina). Deionized water was used.

### 2.2 Obtaining apple by-products

The raw material was processed at the Food Pilot Plant of Departamento de Industrias (Facultad de Ciencias Exactas y Naturales, Universidad de Buenos Aires). The apples were sanitized with an aqueous solution of sodium hypochlorite, peeled, cored, and cut into 12 pieces with a fruit peeling and corer machine (Zhengzhou Xinshijia Machinery Equipment Co., Model QPJ-APP10). They were subsequently immersed in citric acid aqueous solution (0.5 g/100 mL) and ascorbic acid aqueous solution (2.3 g/100 mL) in order to delay their browning during the juice extraction process by pressure (Metalúrgica VZ, Córdoba, Argentina). After obtaining the juice, the by-products (peel remaining from peeling and pulp remaining from pressing) were washed with water and ethanol 96% (v/v) to eliminate intrinsic sugars from the apple tissue that could interfere with the handling of the material during dehydration and affect the obtaining of fractions enriched in pectin.

To obtain the material enriched in cell wall components (CWM) from both the pulp (CWM-Pulp) and the peel (CWM-Peel), the washed material was dehydrated in a convection dryer at 70°C and with an air speed of 6 m/s until obtaining an aqueous activity between 0.3 and 0.4, thus ensuring its stability during storage (Clemenson et al., 2012). A 212–710 µm powder rich in the cell wall material (CWM) was obtained through grinding with a domestic appliance (Wemir E909, Buenos Aires, Argentina) and sieving with ASTM sieves 25 (standard 710 µm) and 70 (212 µm) (Zonytest, Buenos Aires, Argentina). The sieving was performed by means of a vibrating device (sieve shaker) manufactured by the technical staff of the Departamento de Industrias (Facultad de Ciencias Exactas y Naturales, Universidad de Buenos Aires). The yield was calculated as g of CWM per 100 g of by-product.

### 2.3 Characterization of the CWM

To evaluate the cellulose and lignin content, acid hydrolyses were carried out under the action of sulfuric acid and the application of heat, according to Ng et al. (1998). Specifically, the CWM of each tissue was subjected to hydrolysis with sulfuric acid (72 g/100 g) for 3 hours at room temperature. This suspension was then brought to a sulfuric acid concentration of 1 mol/L by adding a sufficient amount of MilliQ water, and the samples were heated at 100°C for 2.5 h. Once cooled, they were centrifuged for 10 min at 10,000 rpm. The residue was carefully separated from the supernatant, washed with deionized water, centrifuged for 10 min at 10,000 rpm, and finally lyophilized. The dry residue obtained was weighed and reported as lignin. A second procedure applied to the CWMs consisted of their hydrolysis with a sulfuric acid solution with a concentration of 1 mol/L at 100°C for 2.5 h. Then, the same steps described above were followed, and the lyophilized residue obtained corresponded to cellulose + lignin. Both hydrolyses were performed in triplicate. Non-cellulosic carbohydrates, uronic acids, and protein content were determined in triplicate in the supernatants with the methods described by Dubois et al. (1956), Filisetti-Cozzi and Carpita (1991) and Lowry et al. (1951), respectively (Santo Domingo et al., 2015).

The moisture content of the powders was determined in triplicate by means of an infrared scale (Moisture Analyzer MB45Ohaus Corporation, New Jersey, USA), using a 0.5-g sample. Water activity was measured in duplicate at 25°C in a Decagon AquaLab (Series 3 Water Activity Meter, Pullman, WA, USA).

### 2.4 Obtaining pectin fractions

#### 2.4.1 NADES production

In the first stage, the production of natural eutectic solvents (NADES) was optimized. Two NADES formulations were selected (N1 and N2). N1 consisted of a mixture of citric acid, glucose, and Milli Q water in a 1:1:3 M ratio, respectively (Benvenutti et al., 2020). N2 was obtained by mixing lactic acid, glucose, and Milli Q water in a molar ratio of 5:1:3, respectively (Dai et al., 2013). An aqueous solution acidified with citric acid (CA) to pH 2.3 was also prepared with the objective of comparing the characteristics of the pectic fractions isolated with the NADES solvents to that isolated with citric acid.

#### 2.4.2 Extraction of pectic fractions

Heat assistance (80°C) was used to extract the fractions enriched in pectin (PF), under stirring at 300 rpm (Velp Scientifica, Usmate, Italy), for 1 hour, both in CWM of pulp with CA, N1, and N2 (PF1, PF2 and PF3) and in CWM of peel with CA, N1, and N2 (PF4, PF5, and PF6), using a CWM–extractant ratio of 1:30 (m/v).

In particular, the extractions carried out with the NADES followed two different procedures:

i) Direct extraction from CWM with NADES. Given that, in the previously mentioned conditions, the extraction produced a very low or null yield of fractions enriched in pectins, it was decided to perform a pretreatment procedure (Chen and Lahaye, 2021) with the selected NADES, followed by an aqueous extraction of the pectins (see item ii).
ii) Two-stage extraction, a first pretreatment stage, where the CWM was put in contact with the NADES under the aforementioned conditions (80°C, 1 h), and a second treatment stage where three parts by volume of Milli Q deionized water were added with respect to the NADES, and the solution was subjected to the same conditions as the first stage.

Subsequently, both the solutions obtained from the extractions using NADES (second procedure) and the aqueous citric acid solution were centrifuged at 4°C at 10,000 rpm (Centrifuge 58.4R Eppendorf, Taufkirchen, Germany) for 20 min. Two volumes of 96% (v/v) ethanol were added to the supernatant obtained to allow the enrichment of the pectin fractions. The precipitate obtained was filtered through muslin cloth, and residual ethanol was eliminated by evaporation under a lab hood. The fractions obtained with N1 and N2 required one washing with MilliQ water and another with 96% (v/v) ethanol in a 2:1 ratio (v/m, ethanol-fraction) and subsequent centrifugation to eliminate glucose prior to lyophilization.

All fractions thus obtained were lyophilized after removing the ethanol to obtain fractions with an a_w_ lower than 0.3–0.4 (Clemenson et al., 2012). The water was frozen (−18°C) and then sublimated in a Pennsalt freeze dryer (Pennsalt, Philadelphia, USA) at a chamber pressure of 100 µm and shelf temperature of 25°C. The powder obtained was milled in a domestic blade mill (DeLonghi, Buenos Aires, Argentina), packaged in polyvinyl chloride/polyvinylidene chloride copolymer bags, and stored in a freezer at temperatures below −10°C for later characterization.

#### 2.4.3 Ultrasound-assisted pectin extraction

A preliminary study was carried out for the extraction of pectic fractions using N2 and applying ultrasound assistance in the pretreatment, with the aim of exploring the usefulness of this assistance to increase the extraction yield. The selection of N2 for this study was linked to the interesting results obtained with this NADES in the extraction without US assistance (Section 3.6). For this purpose, 1 g of CWM-Pulp or CWM-Peel was contacted with 30 mL of N2 and assisted with ultrasonic equipment (Vibracell^®^, 750 W, Sonics Materials Inc, United States. Constant frequency: 20 Hz) using wave amplitudes of 20% or 50% and applying pulses (5 s on and 5 s off). A solid tip (Sonics and Materials, USA), with a tip diameter of 13 mm, was used. The pretreatment time was determined by the temperature, which, due to limitations imposed by the equipment, should not exceed 70°C. Then, the treatment consisting of extraction with water was applied by means of the addition of three parts by volume of MilliQ water at 80°C to the sonicated solution, under stirring for 30 min or 60 min. After this treatment, the same procedure was followed as in the extraction without US assistance, as explained in item 2.4.2.

### 2.5 Characterization of the pectin-enriched fractions

Yield was calculated as g of fraction isolated per 100 g of CWM used.

#### 2.5.1 Chemical analysis

Aqueous solutions were prepared with each fraction, and the content of total carbohydrates, uronic acids (UA), proteins, and methanol was determined. Deionized (Milli-QTM) water was used to prepare the reagents for all chemical assays. The methoxylation degree (DM) was calculated as the percent ratio between moles of methanol and moles of UA in the analyzed sample. All determinations were performed according to Santo Domingo et al. (2015) and in triplicate. The neutral sugar content was determined from the carbohydrate and UA content.

The analysis of neutral sugars was carried out using gas chromatography after hydrolysis with 2 M trifluoroacetic acid. The monosaccharides obtained were reduced with NaBH_4_ and derivatized using pyridine. These compounds were identified using the corresponding standards and a gas chromatograph (Hewlett Packard 5890A) with a capillary column (30 m, 0.25 mm i.d. 0.20 μm) equipped with a flame ionization detector (FID) operated at 240°C. The injection gas was nitrogen, and the internal standard for quantification of the monosaccharides was inositol (Salato et al., 2013; Hammi et al., 2018).

#### 2.5.2 Differential scanning calorimetry (DSC)

DSC analysis was performed using a Mettler instrument (Modelo 822, Mettler Toledo AG, Greifensee, Suiza). A 10–15 mg of dried pectin was placed in a standard aluminum crucible, which was then sealed. The crucible was heated from −20°C to 350°C at a rate of 10°C/min in a dynamic inert nitrogen environment. An empty standard aluminum crucible was employed as a reference at the same time (Wang et al., 2014; Wani and Uppaluri, 2023). The thermograms were evaluated using a Mettler Stare Thermal Analysis System version 3.1 (Mettler Toledo, Schwerzenbach, Switzerland).

#### 2.5.3 Thermogravimetric analysis

Thermogravimetric analysis (TGA) was carried out on a simultaneous TG–DSC/DTA TA thermal analyzer (Modelo SDT Q600, TA Instruments, New Castle, DE, USA) equipped with a nitrogen flow device and a data acquisition system. The samples were heat-treated under nitrogen flow from room temperature to 500°C at a heating rate of 10°C/min using 10 mg of sample mass. Thermal stability was measured by TGA (% weight loss vs. temperature) and derived TG (DTG). The maximum degradation rate (T peak: T_p_) was determined from the peak of the derivative curves (Wani and Uppaluri, 2023) using Thermal Advantage software (TA Instruments, New Castle, DE, USA). Percentage weight loss (Δ m) was calculated by the percentage weight difference between T onset (T_on_) and T offset (T_off_).

#### 2.5.4 Fourier transform infrared spectroscopy (FT-IR)

The IR spectra of samples were recorded with a Nicolet 8700 FT-IR (Thermo Fisher Scientific, MA, USA) equipped with a DTGS detector equipped with Smart Orbit. The KBr pellet method was used. For this purpose, the powdered pectin was mixed with potassium bromide (KBr) and compressed into a pellet, and the spectrum was recorded in the range of 4,000 cm^−1^ to 400 cm^−1^ and with 4 cm^−1^ resolution with OMNIC software version 7.3 (Thermo Fisher Scientific, Waltham, MA, USA).

#### 2.5.5 Color

The color was evaluated in triplicate using a colorimeter (Minolta Co. Ltd., Osaka, Japan) with D65 illuminant and a 2° observer angle. Each sample was placed on a white tile, and the color was recorded through the chromatic coordinates L*(0, black to 100, white), a* (+ a red color; − a green color), and b* (+b yellow color; −b blue color) from the CIELab space (Otálora et al., 2020).

#### 2.5.6 Rheological analysis

Rheological characterization was performed for 1 g/100 mL and 2 g/100 mL aqueous systems with or without sucrose and acidification (citric acid, 50 g/100 mL) using a Modular Compact Rheometer (Model MCR 102e, Anton Paar, Seiersberg, Graz-Umgebung, Austria) equipped with a cone and plate (CP40-2) geometry (diameter: 40 mm, angle: 2°). A gap size of 0.171 mm was set. The temperature was kept constant at 20.0°C using a Peltier system.

Sample flow behavior was evaluated in duplicate through rotational experiments by recording viscosity (η) and stress (*τ*) as a function of shear rate ( ẏ ) (0–300 s^−1^). Each data point was recorded at a steady state. Ostwald’s power law and the Herschel–Bulkley model were used for data fitting.

Ostwald model:

$$\tau =K{\left(ẏ\right)}^{n}.$$

Herschel–Bulkley model:

$$\tau =\tau_{0}+K{\left(ẏ\right)}^{n},$$

where τ is the shear stress (Pa), τ_0_ is the minimum shear stress (Pa), K is the consistency coefficient (Pa. s^n^), n, dimensionless parameter, is the flow behavior index (n < 1 pseudoplastic fluid), and ẏ is the shear rate (s^−1^).

### 2.6 Statistical analysis

Non-linear fits and statistical evaluations were made using the Prism 5 software (GraphPad, California, USA). The statistical significance of the differences was carried out using ANOVA with a level, α, of 0.05. The Tukey test was used as an “*a posteriori test*” (Sokal and Rohlf, 2000).

## 3 Results and discussion

### 3.1 CWM composition

The apple CWM-Pulp and CWM-Peel showed a peel yield of 17.8 g/100 g and a pulp yield of 6.6 g/100 g, with moisture contents of 5.5 g/100 g and 7.5 g/100 g and water activities (a_w_) of 0.29 and 0.37, respectively (Table 1).

**TABLE 1:** Yield, water activity (A_W_), and chemical composition of the cell wall material (CWM) of apple pulp and peel.

Yield and AW	CWM-Pulp (g/100 g wet basis)	CWM-Peel (g/100 g wet basis)
Yield	6.6 ± 0.6^a^	17.8 ± 0,7^b^
A_W_	0.37 ± 0.01^a^	0.29 ± 0.01^b^

Composition	CWM-Pulp	CWM-Peel
Total non-cellulosic carbohydrates	57.8 ± 1.0^a^	40.4 ± 1.4^b^
Uronic acids	11.4 ± 0.3^a^	10.1 ± 0.2^b^
Cellulose	7.8 ± 0.8^a^	12.7 ± 0.5^b^
Lignin	14.4 ± 0.7^a^	30.3 ± 0.6^b^
Protein	8.5 ± 0.7^a^	3.2 ± 0.3^b^
Moisture	7.5 ± 0.1^a^	5.5 ± 0.8^b^

Average and standard deviation are provided. Different letters in the same row indicate significant differences (*p* < 0.05).

The non-cellulosic carbohydrate concentration was determined using a glucose standard curve.

The CWM-Pulp showed a non-cellulosic carbohydrate content (57.8 g/100 g) higher than peel (40.4 g/100 g). Lignin and cellulose constitute the main structural components of the plant cell wall (George et al., 2020). In the CWM-Peel, a content of 30.3 g/100 g of lignin and 12.7 g/100 g of cellulose was determined. These data were significantly higher than those of the CWM-Pulp. This trend is consistent with the protective function of the fruit that the peel fulfills, lignin being primarily responsible for giving rigidity to the cell wall, making it resistant to impacts and bending (Escudero Álvarez and González Sánchez, 2006). The UA and protein contents were 11.4 g/100 g for the UA and 8.5 g/100 g for proteins in the CWM-Pulp, while for CWM-Peel, the values were 10.1 g/100 g and 3.2 g/100 g, respectively.

### 3.2 Pectic fraction composition

As seen in Table 2, pectic fractions with low moisture content (6.1–9.0 g/100 g) and a_w_ values between 0.24 and 0.45 were obtained, thus ensuring their stability against deterioration during storage (Clemenson et al., 2012). The fractions extracted with NADES were less luminous and showed an intensification of red colors and a decrease in yellows compared to those extracted with citric acid. This could affect their potential for application in food products.

**TABLE 2:** Yield, water activity (A_W_), chemical composition, and color of pectin-enriched fractions isolated from pulp and peel CWMs through different extraction methods.

Yield and AW	Fraction					
	PF1	PF2	PF3	PF4	PF5	PF6
Yield	12.2 ± 0.8^cA^	2.5 ± 0.5^aA^	5.1 ± 0.4^bA^	9.6 ± 1.8^bA^	4.3 ± 0.9^aB^	3.9 ± 0.9^aA^
A_W_	0.31 ± 0.01^aB^	0.38 ± 0.05^aA^	0.37 ± 0.01^aA^	0.24 ± 0.01^aA^	0.45 ± 0.03^bB^	0.28 ± 0.10^aA^

Composition	PF1	PF2	PF3	PF4	PF5	PF6
Total carbohydrates	83.7 ± 0.4^cA^	80.7 ± 0.9^bA^	77.1 ± 1.6^aA^	84.0 ± 1.8^bA^	77.2 ± 0.4^aA^	76.1 ± 0.8^aA^
Uronic acids	50.7 ± 0.1^aA^	50.1 ± 0.9^aA^	62.8 ± 1.0^bB^	51.0 ± 1.1^aA^	50.9 ± 0.8^aA^	58.1 ± 0.8^bA^
Neutral sugar (*)	33.06	30.08	14.28	32.92	26.33	17.20
Protein	3.4 ± 0.4^aB^	5.3 ± 0.1^bA^	7.9 ± 0.3^cA^	3.2 ± 0.3^aA^	9.1 ± 0.3^bB^	11.1 ± 0.4^cB^
Degree of methoxylation	73 ± 2^bA^	71 ± 3^bB^	53 ± 4^aA^	73 ± 1^bA^	59 ± 2^aA^	54 ± 3^aA^
Moisture	6.1 ± 0.1^aA^	7.5 ± 0.3^aA^	9.0 ± 0.4^bB^	6.3 ± 0.6^aA^	8.1 ± 0.4^bA^	7.0 ± 1.4^aA^

Color	PF1	PF2	PF3	PF4	PF5	PF6
L*	56.9 ± 0.9^cA^	42.5 ± 1.1^bB^	32.7 ± 1.4^aA^	62.3 ± 0.1^cB^	29.4 ± 0.3^aA^	33.9 ± 1.5^bA^
a*	7.0 ± 0.3^aA^	9.1 ± 0.1^bB^	10.8 ± 0.4^cC^	6.2 ± 0.1^aA^	8.4 ± 0.2^bB^	10.8 ± 0.6^cC^
b*	30.6 ± 0.4^cB^	21.4 ± 0.5^bB^	19.1 ± 0.3^aA^	23.6 ± 0.1^cA^	14.4 ± 1.3^aA^	19.1 ± 0.8^bA^

PF1, PF2, and PF3 are fractions isolated from CWM-Pulp with CA, N1, and N2, respectively. PF4, PF5, and PF6 are fractions isolated from CWM-Peel with CA, N1, and N2, respectively.

Average and standard deviation are provided. Yield is expressed as g/100 g of CWM powder. Total carbohydrates, uronic acids, and protein are expressed as g/100 g of isolated fraction. Degree of methoxylation, DM, is expressed as moles of methanol/100 mol of uronic acids. *Calculated by difference.

Different lowercase letters in the same row indicate significant differences (*p* < 0.05) when PF1, PF2, and PF3 are compared or when PF4, PF5, and PF6 are compared. Different capital letters in the same row indicate significant differences (*p* < 0.05) when PF1 and PF4 or PF2 and PF5 or PF3 and PF6 are compared.

Success in isolating a compound from a plant matrix depends on various factors such as the extraction method, the part of the plant treated, temperature, pressure, time, and external energy delivered (Al Ubeed et al., 2022). With respect to the solvent used, there are specific factors that influence the process, such as the affinity of the compound to be extracted with the solvent. The viscosity of the solvent must also be considered because a high viscosity produces a more difficult contact of the solvent with the tissue. In this investigation, those fractions obtained with CA had a higher yield (9.6–12.2 g/100 g) than those extracted with N1 and N2. The high viscosities of the two latter solvents, as well as the washings necessary to eliminate the remaining glucose in these fractions, might be at least a partial explanation for the trend. At 100 s^−1^ at the working temperature (80°C), N1 presented a viscosity of 137 mPa s and N2 of 4.62 mPa s (Gómez Vargas et al., 2023), while the working temperature of the citric acid solution was approximately 1.1 mPa s (Personal communication). Likewise, it was observed that N2 gave rise to a higher yield than N1 in the fractions extracted from pulp (5.1 g/100 g, N2 vs. 2.5 g/100 g, N1) but not in peel where there were no significant differences (≈4.1 g/100 g).

All fractions had a high content of total non-cellulosic carbohydrates (76.1–84.0 g/100 g) and a protein content with values in the range of 3.2–11.1 g/100 g.)

UAs are an important component of pectin; therefore, their determination is a fundamental step in the analysis of its structure (Dranca et al., 2020). Although a notable increase in UA is observed in the fractions when compared to their CWM of origin, they must be identified as “fractions enriched in pectin” because they fail to reach the 65% (m/m) established by the International Pectin Producers Association (IPPA) to be defined as pectins. Those extracted with N2 allowed a significantly higher extraction of UA (58.1–62.8 g/100 g) than those obtained using N1 (50.1–50.9 g/100 g) or CA (50.7–51.0 g/100 g) as an extractant. In each case, no differences were observed in this content when the tissue used was pulp or peel for each extractant.

The degree of methoxylation (DM), or percentage ratio between the moles of methanol and the moles of GalA, is an important characteristic for the rheological behavior of the extracted pectin fraction (Gerschenson et al., 2021). In the present work, isolated pectins were for high methoxyl content with obtained values greater than 50%. It was observed that those obtained from N2 had a lower DM (53%–54%) than those obtained with N1 (59%–71%) or with CA (73%). The lower pH of the NADES probably determined the lower DM observed for the pectins isolated with them.

In the analysis of the effect of tissue source, significant differences were observed exclusively in those samples extracted with N1 that showed lower DM when they originated from peel. Previous works have reported that extracting pectin from apple gives rise to highly methoxylated polysaccharides using inorganic and organic acids, enzymes, and NADES. In particular, Riyamol and Jeevitha (2024) observed that 16 different NADES based on choline chloride, proline, or betaine as hydrogen bond acceptors produced higher methoxylation degrees for pectins isolated from onion peels than those obtained with conventional acid extraction, but this trend was not observed in the present investigation in which NADES of different composition were used.

#### 3.2.1 Sugar composition

The most important sugars present in the isolated fractions were neutral sugars (NS) and uronic acids (UA) (Table 3). Among the NS are arabinose (Ara), galactose (Gal), xylose (Xyl), glucose (Glc), rhamnose (Rha), and, in smaller quantities, mannose (Man). This composition is similar to that of the fractions isolated from apple bagasse by treatment with citric acid by Marcon et al. (2005). The UA content varied between 51 g/100 g and 58 g/100 g of fraction for those isolated from peel and between 50 g/100 g and 63 g/100 g of fraction for those isolated from pulp (Table 2).

**TABLE 3:** Neutral sugar (NS) composition (moles/100 mol) of the pectin-enriched fractions obtained from the citric acid, NADES 1, and NADES 2 treatment.

NS	Fraction					
	PF1	PF2	PF3	PF4	PF5	PF6
Rha	2.5 ± 0.1^aA^	2.2 ± 0.3^aA^	2.2 ± 0.1^aA^	2.5 ± 0.1^aA^	1.7 ± 0.6^aA^	2.7 ± 1.1^aA^
Fuc	ND	ND	ND	ND	ND	ND
Ara	43.3 ± 0.2^cB^	8.9 ± 1.3^aA^	14.1 ± 1.5^bA^	30.3 ± 1.6^cA^	9.5 ± 0.9^aA^	23.1 ± 0.2^bB^
Xyl	5.5 ± 0.3^aB^	12.6 ± 1.1^cA^	9.3 ± 0.8^bA^	4.0 ± 0.3^aB^	7.9 ± 2.2^bA^	6.1 ± 1.7^bA^
Man	1.7 ± 0.3^bB^	0.2 ± 0.0^aA^	ND	0.9 ± 0.1^cA^	0.45 ± 0.07^bB^	0.25 ± 0.07^a^
Gal	4.8 ± 0.1^aA^	5.1 ± 0.7^aA^	7.2 ± 2.5^aA^	5.7 ± 0.4^bB^	4.5 ± 0.4^aA^	7.4 ± 1.6^bA^
Glc	42.3 ± 0.3^aA^	71.2 ± 0.8^cA^	67.2 ± 1.7^bA^	56.5 ± 2.4^aB^	76.1 ± 1.7^bB^	60.6 ± 4.3^aA^

PF1, PF2, and PF3 are fractions isolated from CWM-Pulp with CA, N1, and N2, respectively. PF4, PF5, and PF6 are fractions isolated from CWM-Peel with CA, N1, and N2, respectively. Average and standard deviation are provided. Different lowercase letters indicate significant differences (*p* < 0.05) for each neutral sugar content for pulp-isolated fractions (PF1, PF2, PF3) or peel-isolated fractions (PF4, PF5, PF6). Different capital letters indicate significant differences (*p* < 0.05) for each neutral sugar content between fractions isolated with the same extractant from CWM-Pulp and CWM-Peel (PF1 vs. PF4; PF2 vs. PF5; PF3 vs. PF6). Abbreviations: Rha: Rhamnose; Fuc: Fucose; Ara: Arabinose; Xyl: Xylose; Man: Mannose; Gal: Galactose; Glc: Glucose. ND: not detected.

The Rha content in the fractions was between 1.7 mol/100 mol and 2.7 mol/100 mol of neutral sugar (NS) in the fractions. The Ara contents vary between 8.9 mol/100 mol and 43.3 mol/100 mol, with the highest values observed for the fractions obtained from the extraction with CA from pulp (43.3 mol/100 mol) or from peel (30.3 mol/100 mol), followed by the fractions obtained by extraction with N2 from peel (23.1 mol/100 mol) or from pulp (14.1 mol/100 mol). The lowest values are observed for the fractions isolated with N1. Ara is a neutral sugar that is very sensitive to degradation in the extractive process, so the low pH of NADES (≈1) probably affects its integrity. Gal content ranged between 4.5 mol/100 mol and 7.4 mol/100 mol, and Xyl presented values between 4.0 mol/100 mol and 12.6 mol/100 mol of sugar in the fraction. A high content of Glu was observed in all fractions, which is not expected given that these samples are fundamentally pectins. This glucose could be due to the extraction of non-pectic polysaccharides such as galactoglucomannans, or it could be the residue of soluble sugars that were not completely removed during the isolation of the pectic fractions.

The presence of UA, Rha, Ara, and Gal shows the occurrence of pectic material in these fractions. The presence of Xyl at levels of the order of Gal suggests the presence of pectic xylogalacturonans. The contribution of mannose was low, which would show a low contribution of hemicellulosic mannans to these fractions. It is interesting to observe that NADES gave rise to fractions with less mannose than the fractions extracted with citric acid.

Several ratios can be calculated from the sugar content of the fractions (Houben et al., 2011; Denman and Morris, 2015), which allow for analyzing the structure of the pectin obtained at the polymeric level. They will be discussed below based on the results reported in Table 4.

- The ratio of UA with respect to the neutral sugars found in the side chains is a measure of the linearity of the pectin and is calculated as: [UA/(Fuc + Rha + Ara + Gal + Xyl)]. It is observed that the greatest linearity is presented by the fraction extracted from pulp with N2 followed by the fraction extracted from peel with the same solvent. The fraction extracted from pulp or peel with CA presents the lowest linearity.
- The ratio between UA and Rha (UA/Rha) allows evaluation of the contribution of RG to the pectic population and presents values between 58.73 and 196.20. The highest values are observed for fractions obtained from pulp and peel extracted with N2, and the lowest values correspond to pulp and peel extracted with CA. The higher values obtained indicate a low contribution of RG to the pectin population when the extraction was performed with N2 and N1.
- The ratio of the sugar content of the side chains of RG-I with respect to Rha turns is a measure of the length of the RG-I branches: (Ara + Gal)/Rha. The largest values are observed for pulp and peel extracted with citric acid, followed by peel extracted with N2 and pulp extracted with N2.
- To evaluate the purity of the extracted product (relative richness of pectin components in relation to other extracted compounds), the ratio (Rha + Ara + Gal + Xyl + GalA)/(Glc + Man) can be calculated. Our data show that the highest value is presented by the fraction extracted from pulp with N2 followed by the fraction extracted from peel with the same green solvent.
- Based on the calculation of the relationships between UA and Ara, Gal and Ara, and Rha and Ara, the effect of the process conditions can be evaluated through the decrease in the presence of arabinose, which corresponds to an increase in these ratios. Severe treatment conditions during production can produce the hydrolysis of the arabinose side chains due to their lability (Buergy et al., 2021). It is observed that these ratios show higher values in fractions obtained from pulp or peel extracted with N1 or N2.
- Homogalacturonan (HG) and RG-I are important regions of pectin. The percentage of RG-I can be calculated as twice the molar content of Rha plus that of Gal and Ara, with the highest values being those of the pulp or peel fractions isolated with CA followed by that isolated from peel with N2. In turn, the difference between UA and Rha allows calculating the percentage of HG, which shows values between 57.99 and 80.78, with the highest values being those obtained for fractions extracted from pulp or peel with N2 and the lowest values being those obtained for fractions of both tissues treated with CA. The ratio between HG and RG-I allows us to see the relative richness of these two regions. The results show that the pulp extracted with N2 and the peel extracted with N1 have the highest relative richness in HG and that the fractions extracted from pulp and peel with citric acid have the highest relative richness of RG-I.

**TABLE 4:** Sugar molar ratios for pectin-enriched fractions isolated from apple pulp and peel.

Ratio fraction	Linearity	UA/Rha	(Ara + Gal)/Rha	Purity	UA/Ara	Gal/Ara	Rha/Ara	%HG	%RGI	HG/RG-I
PF1	2.57	58.73	19.61	4.54	3.33	0.11	0.06	57.99	21.71	2.67
PF2	5.61	73.16	6.34	2.66	18.02	0.57	0.25	60.83	7.03	8.65
PF3	13.22	196.20	9.64	6.90	30.72	0.51	0.16	80.78	4.82	16.78
PF4	3.51	59.61	14.36	3.34	4.93	0.19	0.08	58.86	16.43	3.58
PF5	8.17	116.35	8.42	2.81	20.32	0.47	0.17	65.18	5.89	11.07
PF6	8.49	125.57	11.49	6.12	14.44	0.32	0.11	76.28	8.26	9.23
USPFA	7.50	131.14	12.90	6.37	12.72	0.25	0.10	75.67	8.66	8.74
USPFB	10.80	208.03	14.37	11.38	17.45	0.21	0.08	83.73	6.62	12.64
USPFC	5.99	111.96	16.00	5.47	8.08	0.16	0.07	71.80	11.65	6.16
USPFD	4.63	68.13	11.75	8.29	7.09	0.22	0.10	72.32	14.82	4.88

PF1, PF2, and PF3 are fractions isolated from CWM-Pulp with CA, N1, and N2, respectively. PF4, PF5, and PF6 are fractions isolated from CWM-Peel with CA, N1, and N2, respectively.

USPFA and USPFB are fractions isolated from CWM-Pulp with N2, with ultrasound assistance using amplitudes of 20% and 50%, respectively. USPFC and USPFD are fractions isolated from CWM-Peel with N2, with ultrasound assistance using amplitudes of 20% and 50%, respectively.

It can be concluded that the extraction of pectin from apple tissues is very sensitive to the extraction conditions and the extracted tissue. The fractions extracted from pulp and peel with citric acid have less linearity and longer branches. The decrease in pH due to treatment with the chosen NADES solvents led to a significant loss of arabinose when extracted from pulp. These extraction conditions could affect the molecular mass, rheological behavior, and functional properties.

### 3.3 FT-IR

**FIGURE 1:**
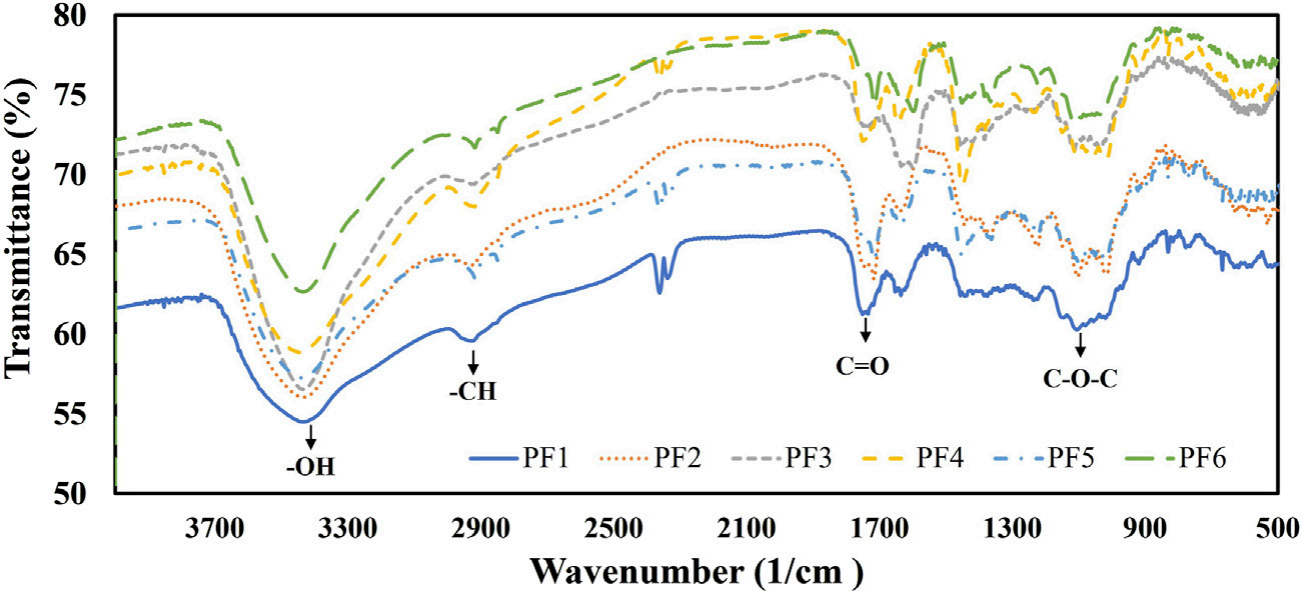
FT-IR spectra of pectin-enriched fractions isolated from CWMs of apple pulp and peel by different extraction methods.

Figure 1 shows the FT-IR spectra of the pectic fractions obtained. A change in intensity was observed in the bands depending on the residue (pulp or peel) and the extractant used (CA, N1, or N2).

The band observed at ≈3,400 cm^−1^ corresponds to the stretching vibration of the -OH groups of galacturonic acid (Zheng et al., 2021). In the different fractions, this band was observed between 3,434 cm^−1^ and 3,437 cm^−1^. The CH stretching vibrations of the CH2 groups are responsible for generating the small band at ≈2,900 cm^−1^ (Xu et al., 2022). For fractions isolated from CWM-Pulp, bands were found between 2,927 cm^−1^ and 2,936 cm^−1^. In the fractions obtained from CWM-Peel, the bands were observed at 2,917–2,919 cm^−1^. Two bands were found for all the fractions obtained from the peel for all the extractants used, one at approximately 2,920 cm^−1^ and the other at 2,850 cm^−1^. The band at approximately 2,850 cm^−1^ is due to remnants of the plant cuticle, such as cutin, waxes, and cutan, and is determined by the symmetrical stretching vibrations of their CH2 groups (Heredia-Guerrero et al., 2014).

The band at ≈1730 cm^−1^ can be ascribed to the vibration of the carboxyl groups esterified with methyl (COO-R). In this band, a shift was observed according to the extracting solvent used. In the case of CA, the wavenumber was 1750 cm^−1^. In the case of N1, it occurred at approximately 1715–1716 cm^−1^, and for N2, it occurred at 1723–1727 cm^−1^. The band observed between 1,632 cm^−1^ and 1,638 cm^−1^ is caused by the asymmetric stretching of the non-esterified carboxylate ions (COO-) to which the band that occurs at approximately ≈1,400 cm^−1^ (carboxylate symmetric stretching band) is also attributed (Koziol et al., 2022). This last band was only detected in PF4 and PF5 with values of 1,448 cm^−1^ and 1,451 cm^−1^, respectively.

In the isolated fractions, the weak band observed at approximately 1,230 cm^−1^ is attributed to the vibrations of the pectin side chains. Bands with wave numbers at approximately 1,105 cm^−1^ and 1,014 cm^−1^ were detected, allowing the identification of pectic polysaccharides rich in uronic acids (Alonso-Simón et al., 2004). The bands of medium or low intensity observed below 920 cm^−1^ in the “fingerprint” region are fundamentally attributed to vibrations of the C–O–C bridges typical of polysaccharides (Koziol et al., 2022).

The FT-IR spectra recorded were characteristic of the typical polygalacturonic acid backbone of pectins.

### 3.4 Thermal analysis

#### 3.4.1 DSC

**FIGURE 2:**
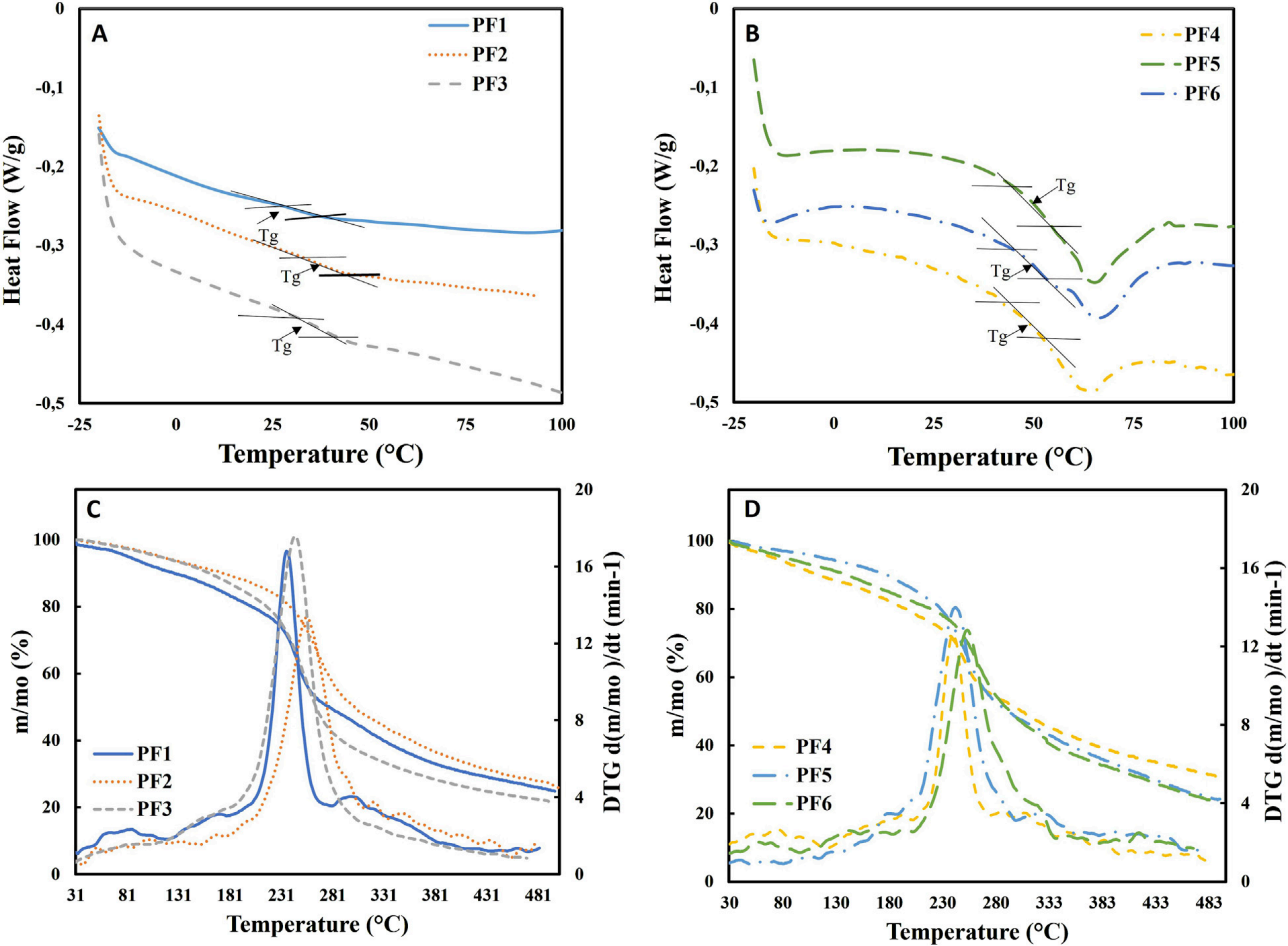
DSC thermograms of pectin-enriched fractions isolated from CWMs of apple **(A)** pulp and **(B)** peel. TGA and DTG curves of pectin-enriched fractions isolated from CWMs of apple **(C)** pulp and **(D)** peel.

The DSC curve describes the changes in the enthalpy of the reaction during the degradation of the sample, allowing the recording of endo- or exothermic reactions in the working temperature range, as well as second-order transitions such as glass transitions (Einhorn-Stoll et al., 2007). Figure 2 (Panels A and B) shows the results obtained for samples PF1, PF2, and PF3 compared to PF4, PF5, and PF6. This figure, as well as Table 5, show that the pectins isolated with CA and with NADES presented Tgs in the range 38–43°C (Tp) when they were obtained from CW-Pulp and 50–53°C (Tp) when the origin was CW-Peel. The specific heats have higher values for the isolated peel fractions. In particular, samples PF5 and PF6 show the highest Tg values. Similar values to those obtained in this work have been reported for pectins obtained from apple bagasse by Zlatanović et al. (2019).

**TABLE 5:** Parameters of the thermal analysis of the pectin-enriched fractions.

	DSC				TGA/DTG					
Fraction	T_on_ (°C)	T_p_ (°C)	T_off_ (°C)	cp (J g^−1^ K^−1^)	T_on_ (°C)_DTG_	T_p_ (°C)_DTG_	T_off_ (°C)_DTG_	ΔT (°C)_DTG_	Δm (%)*	Δm/ΔT (%°C^−1^)
PF1	29.98	38.06	38.25	0.130	206	244	270	64	27.9	0.43
PF2	32.08	40.97	43.40	0.144	200	265	309	109	39.4	0.36
PF3	33.41	43.09	45.17	0.177	193	261	300	107	46.8	0.44
PF4	45.43	50.04	54.53	0.322	206	249	274	68	22.8	0.33
PF5	48.18	53.13	57.19	0.380	209	260	305	96	37.6	0.39
PF6	47.67	51.20	54.28	0.228	204	264	342	138	42.9	0.31

Ton/Tp/Toff: extrapolated onset, peak, and offset temperature of the DSC or DTG curves. cp: specific heat. ΔT: peak width of the DTG curve. Δm: total mass loss between T_on_ and T_off_ of the DTG curve. *: with reference to m_0_.

It is important to note that the literature reports Tg values for pectin ranging from 11°C (Aguilera et al., 1998) to 79°C (Ruano et al., 2020). This diversity of values is determined by the different origins of the pectins (plant and tissue), their different moisture contents (Basu et al., 2013), and their composition and chemical structure, which are affected by the hydrolysis reactions that this macromolecule can suffer during its production and storage (Ruano et al., 2020; Einhorn-Stoll et al., 2015).

The fractions obtained from peel (PF4, PF5, and PF6) presented a broad endothermic peak at ≈65°C that could be attributed to changes in the state of the cutin present in the peel (Aggarwal, 2001). Analogous results were found by Aguilera et al. (1998) for freeze-dried and dehydrated tissue of Granny Smith apples, reporting an endothermic peak at 50–70°C.

#### 3.4.2 TGA/DTG

A thermogravimetry analysis (TGA) was carried out to investigate the thermal stability of the obtained pectins and determine any significant differences in their decomposition. Figure 2 (Panels C and D) shows the TGA and DTG curves. The TGA curve reports weight loss during heating. The DTG curve is the first derivative of the TGA curve and provides information on the degradation rate (Einhorn-Stoll et al., 2007; Einhorn-Stoll and Kunzek, 2009).

The TGA curves show that the six samples presented a similar shape, differentiating three stages, as has been reported in other investigations (Zhou et al., 2023; Hu et al., 2020). A first slight decrease in mass, around 100°C, was probably caused by the evaporation of the water sorbed in the pectins, and a much more pronounced decrease between 200°C and 350°C was caused by the decomposition of the polysaccharide chains. The uronic acid chains probably underwent thermal decomposition and then decarboxylation of the acid groups, as well as an onset of carbonization. Finally, between 350°C and 400°C, a slow decrease in weight was again observed, possibly caused by the thermal decomposition of the charred material.

By extrapolating the degradation temperatures, it was possible to obtain the start and end temperatures of degradation for each fraction, observing that the six samples began their degradation reaction at temperatures between 193°C and 209°C. The degradation of the six pectins showed, in general, a single peak in the DTG curves, in the range of 200–350°C (Figures 2C, D; Table 5), which corresponded to the decomposition of the pectin chains, with a rapid mass loss ranging between 22.8% and 46.8%. Einhorn-Stoll et al. (2007) reported values of the order of 50% for pectin extracted from citrus fruits. The samples extracted with NADES, PF2, and PF3 (pulp), and PF5 and PF6 (peel) presented the highest peak temperatures (Tp) at the maximum rate of weight loss, suggesting that they are the most thermally stable fractions of the six obtained. Regarding the total mass loss between T_on_ and T_off_ of the DTG curve, the lowest value corresponded to the pectin fractions PF1 and PF4 extracted with CA. The lowest total mass loss per unit of temperature in the T_on_–T_off_ range was observed for PF6 extracted from CWM-Peel with N2.

### 3.5 Rheological properties

Knowledge of rheological properties is essential for the adequate formulation of food products as well as for the design of their preservation processes. Therefore, the rheological properties of pectin-enriched fractions were investigated to evaluate their functionality. For this, aqueous solutions of 1 g/100 mL and 2 g/100 mL were evaluated with and without sucrose and acidification. Note that, for the formation of a gel, high methoxyl pectins require the addition of sucrose and acid that favor the establishment of hydrophobic interactions and hydrogen bonds (Zhao et al., 2020). Sucrose induces the heterogeneous distribution of water around the pectin macromolecules, producing self-assembly of the polymer chains, which contributes to the increase in viscosity and could result in macroscopic gelation in an acidic medium.

**FIGURE 3:**
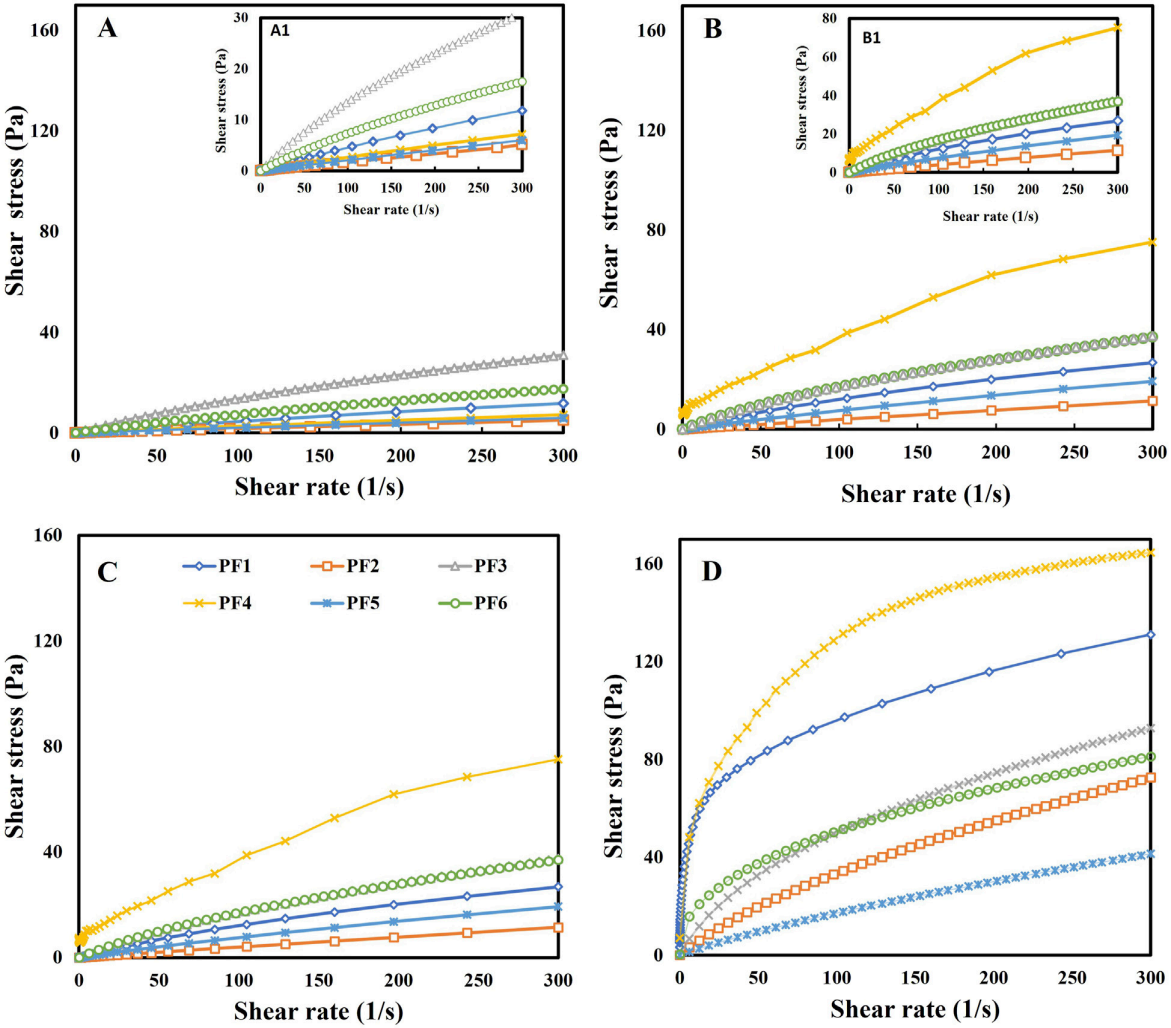
Flow curves at 20°C for aqueous systems of pectin-enriched fractions isolated from CWM of apple pulp and peel. **(A)** 1 g/100 mL without sucrose, **(C)** 2 g/100 mL without sucrose, **(B)** 1 g/100 mL with sucrose, and **(D)** 2 g/100 mL with sucrose.

The results obtained can be observed in Figure 3 and Tables 6, 7. From the flow curves, it can be concluded that all fractions formed viscous solutions that behaved like pseudoplastic fluids because their viscosity decreased as the shear rate increased. This behavior can be attributed to structural changes in the macromolecular solutions due to the increase in the shear stress applied (Dranca et al., 2020). Analogous results were reported by Morales-Contreras et al. (2020) for high methoxyl apple pectin isolated with hydrochloric acid. Fissore et al. (2009) studied the flow behavior of pectins with a high degree of methoxylation, isolated by enzymatic treatment (hemicellulase), from *Cucurbita moschata Duch* ex *Poiret*, observing that their aqueous solutions presented pseudoplastic behavior that was attributed to the structure generated by the high density of interactions established between hydrated macromolecules.

**TABLE 6:** Parameters of Ostwald and Herschel–Bulkley models evaluated at 20°C in 1 g/100 mL aqueous systems of pectin-enriched fractions without or with sucrose addition and acidification.

Fraction	Sucrose/acidification				No sucrose/acidification		
1%	K (Pa s^n^)	n	τ_0_	R^2^	K (Pa s^n^)	n	R^2^
PF1	0.317 ± 0.001^bA^	0.783 ± 0.001^bA^	—	0.999	0.072 ± 0.009^aB^	0.896 ± 0.003^bA^	1
PF2	0.044 ± 0.001^aA^	0.972 ± 0.001^cB^	—	1	0.022 ± 0.003^aB^	0.963 ± 0.004^cA^	1
PF3	0.567 ± 0.002^cA^	0.736 ± 0.001^aA^	—	0.999	0.389 ± 0.008^bB^	0.769 ± 0.001^aA^	0.995
PF4	0.862 ± 0.165^bB^	0.779 ± 0.038^aA^	5.983 ± 0.080	0.994	0.036 ± 0.00^bA^	0.929 ± 0.001^bB^	0.999
PF5	0.129 ± 0.004^aB^	0.879 ± 0.006^bA^	—	1	0.024 ± 0.001^aA^	0.966 ± 0.006^cA^	1
PF6	0.61 ± 0.03^bA^	0.722 ± 0.010^aA^	—	0.999	0.175 ± 0.003^cA^	0.81 ± 0.03^aB^	0.999

Average and standard deviation are provided. PF1, PF2, and PF3 are fractions isolated from CWM-Pulp with CA, N1, and N2, respectively. PF4, PF5, and PF6 are fractions isolated from CWM-Peel with CA, N1, and N2, respectively. Different lowercase letters in the same column indicate significant differences (*p* < 0.05) when PF1, PF2 and PF3 are compared or when PF4, PF5 and PF6 are compared. Different capital letters in the same column indicate significant differences (*p* < 0.05) when PF1 and PF4 or PF2 and PF5 or PF3 and PF6 are compared.

K: consistency coefficient. n: flow behavior index. τ_0_: minimum shear stress.

**TABLE 7:** Parameters of Ostwald and Herschel–Bulkley models evaluated at 20°C in 2 g/100 mL aqueous systems of pectin-enriched fractions without or with sucrose addition and acidification.

Fraction	Sucrose/acidification				No sucrose/acidification		
2%	K (Pa s^n^)	n	τ_0_	R^2^	K (Pa s^n^)	n	R^2^
PF1	25.8 ± 3.2^bA^	0.29 ± 0.01^aA^	—	0.991	3.32 ± 0.04^cB^	0.618 ± 0.001^aA^	0.994
PF2	1.22 ± 0.06^aB^	0.72 ± 0.01^cA^	—	0.996	0.56 ± 0.02^aB^	0.772 ± 0.001^cB^	0.999
PF3	3.35 ± 0.06^aA^	0.584 ± 0.003^bB^	—	0.999	1.64 ± 0.04^bA^	0.663 ± 0.003^bB^	0.998
PF4	32.8 ± 1.9^cA^	0.29 ± 0.01^aA^	—	0.985	1.84 ± 0.02^bA^	0.6307 ± 0.0001^aB^	0.998
PF5	0.40 ± 0.04^aA^	0.81 ± 0.03^cB^	—	0.999	0.129 ± 0.004^aA^	0.879 ± 0.006^bB^	0.999
PF6	5.1 ± 0.2^bB^	0.47 ± 0.01^bA^	5.8 ± 0.8	0.986	2.18 ± 0.02^cB^	0.62 ± 0.01^aA^	0.993

Average and standard deviation are provided. PF1, PF2, and PF3 are fractions isolated from CWM-Pulp with CA, N1, and N2, respectively. PF4, PF5, and PF6 are fractions isolated from CWM-Peel with CA, N1, and N2, respectively. Different lowercase letters in the same column indicate significant differences (*p* < 0.05) when PF1, PF2 and PF3 are compared or when PF4, PF5 and PF6 are compared. Different capital letters in the same column indicate significant differences (*p* < 0.05) when PF1 and PF4 or PF2 and PF5 or PF3 and PF6 are compared.

K: consistency coefficient. n: flow behavior index. τ_0_: minimum shear stress.

The experimental data were analyzed using the Ostwald model and the Herschel–Bulkley model. The results can be seen in Tables 6, 7. In general, the polymeric solutions of the fractions presented a flow behavior that significantly adjusted to the Ostwald model, with the exception of PF4 (1 g/100 mL) and PF6 (2 g/100 mL), both with sucrose and acidification, whose flow behavior significantly adjusted to the Herschel–Bulkley model, presenting a minimum shear stress (τ_0_) of ≈5.9 Pa. This indicates they are interesting hydrocolloids for the formulation of foods due to the possibility of using them as thickeners that also allow controlling the rate of discharge from their containers.

Although all fractions gave rise to aqueous solutions with the behavior of pseudoplastic fluids, significant differences were found in the flow behavior index (n) and the consistency coefficient (K) depending on the technique applied for obtaining the fraction, its concentration, and the presence of sucrose and acidification. In all cases, K increased, and n decreased i) when incorporating sucrose, both at 1 g/100 mL and at 2 g/100 mL, or ii) when the fraction concentration was increased from 1 g/100 mL to 2 g/100 mL in the presence or absence of sucrose. Those fractions isolated with CA and N2 showed, in general, higher K and lower n in the 1 g/100 mL or 2 g/100 mL solutions than those isolated with N1 (Tables 6, 7). This might be linked to the composition of the different isolated fractions and, more specifically, to the characteristics of the macromolecules present in them. Davitadze (2023) reported that for pectins, the content of neutral sugar and, therefore, the shielding of the carboxyl groups by those sugars contributes to the twisting and formation of globular structures by the macromolecules, thus contributing to its rheological behavior. Therefore, prolonged thermal treatments of pectic extracts, which destroy arabinose, hydrogen bonds, and arabinan, galacturonan, and galactan bonds, can affect the rheological behavior.

The fractions PF1 and PF4 at 2 g/100 mL in aqueous solution containing sucrose presented a significantly higher K than the rest (32.8 Pa s^n^ and 25.8 Pa s^n^), thus evidencing interesting properties due to their important potential contribution to the texture of foods. Their low n (0.29) showed an important decrease in viscosity with the increase in shear rate.

The viscosity and flow characteristics of the systems indicate that they can be used as potential food thickeners and stabilizers.

### 3.6 Ultrasound-assisted pectin extraction

The objective of ultrasound pretreatment in the extraction of pectin using NADES was to evaluate whether cavitation increased the yield of the pectin fractions through its action on apple tissues. This evaluation was performed with N2 extraction because the characterization of fractions obtained with this solvent without US pretreatment showed a greater content of UA, higher purity, larger ramifications on Rha, and higher Tg. For all this, and although it is known that US can alter the chemical structure and functionality of pectin, N2 seemed to be the most interesting NADES to be explored. The results obtained are reported below.

The yield when the US pretreatment amplitude was 20% and the solvent treatment duration was 30 min were 13.9 g/100 g CWM-Pulp (USPFA) and 6.4 g/100 g CWM-Peel. Under the same conditions with a US amplitude of 50%, they were 15.1 g/100 g CWM-Pulp (USPFB) and 6.3 g/100 g CWM-Peel.

The yield when the US amplitude in pretreatment was 20% and the treatment duration was 60 min were 8.14 g/100 g CWM-Pulp and 17.9 g/100 g CWM-Peel (USPFC). Under the same conditions, but with a US amplitude of 50%, they were 7.4 g/100 g CWM-Pulp and 13 g/100 g CWM-Peel (USPFD). This indicates that in the case of pulp, at both amplitudes, shorter times (30 min) produced higher yields, but in the case of peel, longer treatment times (60 min) obtained higher yields. Accordingly, work continued with the fractions obtained with the treatments that gave greater yield for each tissue, and their composition was characterized.

The results obtained can be seen in Table 8. The yields even exceeded the values reported in Table 2 for the treatments with CA, which were the highest in the absence of US assistance. It can be seen in Table 8 that in the proposed technique with US, a greater enrichment in UA was obtained (USPFA, USPFB, and USPFD). In particular, the fractions obtained from pulp with values of 70.7 g/100 g for USPFA and 70.6 g/100 g for USPFB could be called pectins as established by the IPPA (International Pectin Producers Association, 2024) because they exceed the value of 65 g/100 g. In contrast, the fraction obtained from peel with US pretreatment of 50% amplitude, although it showed higher UA content (63.6 g/100 g in USPFD) than the fraction obtained with N2 and without the assistance of US (Table 2, Fraction PF6, 58.1 g/100 g), did not reach the minimum established to be considered pectin under the IPPA standard. The values for DM ranged between 68% and 70%, being higher than those observed for PF3 and PF6 (53%–54%) (Table 2).

**TABLE 8:** Yield, water activity (A_W_), and chemical composition of pectin-enriched fractions isolated from pulp and peel CWMs with NADES2 and ultrasound.

Yield and Aw	Fraction			
	USPFA	USPFB	USPFC	USPFD
Yield	13.9	15.1	17.9	13
Aw	0.33 ± 0.01^aA^	0.36 ± 0.01^aA^	0.32 ± 0.01^aA^	0.33 ± 0.01^aA^
Composition				
Total carbohydrates	91.9 ± 1.6^aB^	87.5 ± 3.3^aA^	75.2 ± 1.8^aA^	85.3 ± 0.1^bA^
Uronic acid	70.7 ± 3.87^aB^	70.6 ± 1.90^aB^	55.1 ± 0.90^aA^	63.6 ± 2.56^bA^
Neutral sugar (*)	21.2	12.9	20.1	21.7
Protein	6.2 ± 0.6^aA^	6.3 ± 0.2^aA^	12.1 ± 2.3^aA^	14.53 ± 0.05^aB^
Degree of methoxylation	68 ± 2^aA^	68 ± 3^aA^	70 ± 1^aA^	68 ± 2^aA^

USPFA and USPFB are fractions isolated from CWM-Pulp with N2, with ultrasound assistance using amplitudes of 20% and 50%, respectively. USPFC and USPFD are fractions isolated from CWM-Peel with N2, with ultrasound assistance using amplitudes of 20% and 50%, respectively.

Average and standard deviation are provided. Yield is expressed as g/100 g of CWM powder. Total carbohydrates, uronic acids, and protein are expressed as g/100 g of isolated fraction. Degree of methoxylation, DM, is expressed as moles of methanol/100 mol of uronic acids. *Calculated by difference.

Different lowercase letters in the same row indicate significant differences (*p* < 0.05) between USPFA and USPFB or USPFC and USPFD. Different capital letters in the same row indicate significant differences (*p* < 0.05) between USPFA and USPFC or USPFB and USPFD.

#### 3.6.1 Neutral sugars

Regarding the content of neutral sugars, it can be observed by comparing the information provided in Table 8 with that in Table 2 that the fractions USPFA, USPFC, and USPFD showed a higher content of these compounds than the fractions also obtained with N2 but without ultrasound assistance (PF3 and PF6).

When the data are analyzed for each neutral sugar (Table 9), comparing with FP3 or FP6, as appropriate, a significant decrease in Glu (USPFA, USPFB, USPFC, USPFD), a decrease in Gal (USPFC), a decrease of mannose (USPFC), an increase in Rha (USPFA, USPFB, USPFD), an increase in Fuc (USPFA, USPFB, USPFD) and, in general, an increase in Ara are observed.

**TABLE 9:** Neutral sugar (NS) composition (moles/100 mol) of the pectin-enriched fractions obtained with NADES2 and ultrasound.

NS	Fraction			
	USPFA	USPFB	USPFC	USPFD
Ram	2.5 ± 0.1^aA^	2.6 ± 0.2^aA^	2.4 ± 0.1^aA^	4.0 ± 0.1^bB^
Fuc	0.55 ± 0.07^aB^	0.7 ± 0.1^aA^	ND	0.6 ± 0.2^aA^
Ara	25.3 ± 1.1^aA^	30.4 ± 4.7^aA^	32.6 ± 2.8^aA^	38.9 ± 2.7^aA^
Xil	8.3 ± 0.2^aB^	9.2 ± 0.6^aA^	3.9 ± 0.5^aA^	7.4 ± 0.4^bA^
Man	ND	ND	ND	0.30 ± 0.01^bA^
Gal	6.4 ± 0.8^aA^	6.3 ± 0.5^aA^	5.1 ± 0.5^aA^	8.7 ± 0.1^bB^
Glc	57.2 ± 2.3^aA^	50.7 ± 4.1^aA^	56.2 ± 1.8^bA^	40.2 ± 2.3^aA^

USPFA and USPFB are fractions isolated from CWM-Pulp with N2, with ultrasound assistance using amplitudes of 20% and 50%, respectively. USPFC and USPFD are fractions isolated from CWM-Peel with N2, with ultrasound assistance using amplitudes of 20% and 50%, respectively.

Average and standard deviation are provided. Different lowercase letters indicate significant differences (*p* < 0.05) for each neutral sugar content for pulp-isolated fractions (USPFA, USPFB) or peel-isolated fractions (USPFC, USPFD). Different capital letters indicate significant differences (*p* < 0.05) for each neutral sugar content between fractions isolated with the same amplitude from CWM-Pulp and CWM-Peel (USPFA vs. USPFC; USPFB vs USPFD).

Abbreviations: Rha: Rhamnose; Fuc: Fucose; Ara: Arabinose; Xyl: Xylose; Man: Mannose; Gal: Galactose; Glc: Glucose.

ND: not detected.

When the ratios reported for the US-assisted isolated fractions are compared with those corresponding to PF3 or PF6 (Table 4), as appropriate, the following outcomes are observed:

- A decrease in linearity (USPFA, USPFB, USPFC, USPFD).
- An increase in the length of the Ara + Gal chain in Rha (USPFA, USPFB, USPFC).
- An increase in purity (USPFB, USPFD).
- A decrease in UA/Rha (USPFA, USPFC, USPFD).
- A decrease in UA/Ara (USPFA, USPFB, USPFC, USPFD).
- A decrease in Gal/Ara (USPFA, USPFB, USPFC, USPFD).
- A decrease in Rha/Ara (USPFA, USPFB, USPFC, USPFD).
- A decrease in % HG (USPFA, USPFC, USPFD).
- An increase in RG-I % (USPFA, USPFB, USPFC, USPFD).
- A decrease in HG/RG-I (USPFA, USPFB, USPFC, USPFD).

Therefore, these fractions obtained by US pretreatment followed by solvent treatment at 30 min or 60 min, as appropriate, are, in general, less linear, have longer chains above the branch points, and some have greater purity. The treatment has been less aggressive and, therefore, they have a higher Ara content. They show a decrease in the HG/RG-I ratio, which involves a greater conservation of RG-I in US-assisted extraction.

It is expected that this will determine changes in the rheological behavior, possibly favoring thickening and gelation (Davitadze, 2023) by these fractions. This research is currently being carried out.

Different investigations have reported that the bioactivity of pectin is linked to its structural characteristics, such as the presence of RG-I and arabinogalactan side chains and the composition of rhamnogalacturonan and homogalacturonan (Morris et al., 2013; Wu et al., 2018; Ding and Cui, 2020). Niu et al. (2023) analyzed the anticancer, anti-inflammatory, and anti-oxidation effects, as well as the prebiotic and immunity-enhancing effects of pectin. They concluded that the side chains made up of neutral sugars of this complex macromolecule, which constitute the RG-I of pectin, give it different physiological activities. They also evaluated the effect of side chains on pectin thickening and gelling capacity and concluded that their entanglement and crosslinking provide RG-I with very good emulsifying and gelling properties.

Therefore, it is necessary to further study the promising technique using US assistance and NADES to better determine its effect on pectin structure, bioactivity, and technological properties.

## 4 Conclusion

The production of pectins from apple pulp and peel was studied by using NADES solvents (citric acid, glucose, and water-N1 and lactic acid, glucose, and water-N2) and an aqueous solution of citric acid at 80°C. For the extraction of fractions enriched in pectin (PF), heat assistance (80°C) was used. In particular, the extractions carried out with NADES followed a two-stage extraction: a first pretreatment stage, where the apple tissues were put in contact with the NADES, and a second treatment stage, where three additional volumes of water were added. Additionally, ultrasound pretreatment was evaluated using the NADES with lactic acid.

The extraction of pectin from apple tissues turned out to be very sensitive to the extractive conditions and the tissue used. The fractions extracted from pulp and peel with citric acid gave rise to pectins with less linearity and greater branch lengths than those extracted with NADES. These extraction conditions could then affect the rheological behavior and functional properties.

The fractions obtained showed Tgs values between 38°C and 53°C, with the highest values for those isolated from peel with NADES. Regarding the thermal stability of the fractions, those obtained from pulp and peel with NADES were the most stable.

The aqueous solutions of all fractions showed pseudoplastic behavior. Those fractions isolated with N2 showed, in general, higher K and lower n in the 1 g/100 mL or 2 g/100 mL solutions than those isolated with N1, and this would be linked to the characteristics of the macromolecules present in them. The fractions isolated from peel with citric acid (1 g/100 mL) and with N2 (2 g/100 mL), both with sucrose and acidification, presented a minimum shear stress of 5.9 Pa, which denotes them as interesting hydrocolloids for the formulation of foods, due to the possibility of using them as thickeners that also allow controlling the rate of discharge from their containers.

The fractions isolated with citric acid in a 2 g/100 mL aqueous solution with sucrose presented a significantly higher K than the rest (32.8 Pa s^n^ and 25.8 Pa s^n^) and a lower n (0.29), thus evidencing interesting properties and a potential contribution to the texture of foods. The low n shows a notable decrease in viscosity with the increase in shear rate trend that must be taken into account when defining food formulation when intensive fluid circulation or mixing operations are involved in food processing.

The fractions obtained using US and N2 were found to be less linear, had longer chains on the branch points, and some had higher purity than those isolated without US. The treatment turned out to be less aggressive; therefore, a higher Ara content and greater conservation of the RG-I domain were observed. For all these reasons, the change in structure observed in the pectins isolated with the technique that involves US and N2 could give rise to pectic fractions with interesting bioactivities and improved technological properties. As a consequence, it is necessary to deepen this line of research with the objective of increasing the efficiency of the isolation process and improving the functionality of the fractions.

## Data Availability

The raw data supporting the conclusions of this article will be made available by the authors, without undue reservation.

## References

[B1] Aggarwal P. (2001). Phase transition of apple cuticles: a DSC study. Thermochim. Acta 367-368, 9–13. 10.1016/S0040-6031(00)00701-2

[B2] Aguilera J. M. Cuadros T. R. del Valle J. M. (1998). Differential scanning calorimetry of low-moisture apple products. Carbohydr. Polym. 37 (1), 79–86. 10.1016/S0144-8617(98)00030-7

[B3] Alonso-Simón A. Encina A. E. García-Angulo P. Álvarez J. M. Acebes J. L. (2004). FTIR spectroscopy monitoring of cell wall modifications during the habituation of bean (*Phaseolus vulgaris* L.) callus cultures to Dichlobenil. Plant Sci. 167 (6), 1273–1281. 10.1016/j.plantsci.2004.06.025

[B4] Al Ubeed H. M. S. Bhuyan D. J. Alsherbiny M. A. Basu A. Vuong Q. V. (2022). A comprehensive review on the techniques for extraction of bioactive compounds from medicinal *Cannabis* . Molecules 27 (3), 604. 10.3390/molecules27030604 35163863 PMC8840415

[B5] Antonic B. Jancikova S. Dordevic D. Tremlova B. (2020). Apple pomace as food fortification ingredient: a systematic review and meta‐analysis. J. Food Sci. 85 (10), 2977–2985. 10.1111/1750-3841.15449 32966605

[B6] Basu S. Shivhare U. S. Muley S. (2013). Moisture adsorption isotherms and glass transition temperature of pectin. J. Food Sci. Technol. 50 (3), 585–589. 10.1007/s13197-011-0327-y 24425957 PMC3602573

[B7] Benvenutti L. Sanchez-Camargo A. D. P. Ferreira Zielinski A. A. Salvador Ferreira S. R. (2020). NADES as potential solvents for anthocyanin and pectin extraction from *Myrciaria cauliflora* fruit by-product: *in silico* and experimental approaches for solvent selection. J. Mol. Liq. 315, 113761. 10.1016/j.molliq.2020.113761

[B8] Buergy A. Rolland-Sabaté A. Leca A. Falourd X. Foucat L. Renard C. M. (2021). Pectin degradation accounts for apple tissue fragmentation during thermomechanical-mediated puree production. Food Hydrocoll. 120, 106885. 10.1016/j.foodhyd.2021.106885

[B9] Canteri-Schemin M. H. Ramos Fertonani H. C. Waszczynskyj N. Wosiacki G. (2005). Extraction of pectin from apple pomace. Braz. Archives Biol. Technol. 48 (2), 259–266. 10.1590/S1516-89132005000200013

[B10] Chemat F. Rombaut N. Sicaire A.-G. Meullemiestre A. Fabiano-Tixier A.-S. Abert-Vian M. (2017). Ultrasound assisted extraction of food and natural products. Mechanisms, techniques, combinations, protocols and applications. A review. Ultrason. Sonochemistry 34, 540–560. 10.1016/j.ultsonch.2016.06.035 27773280

[B11] Chen M. Lahaye M. (2021). Natural deep eutectic solvents pretreatment as an aid for pectin extraction from apple pomace. Food Hydrocoll. 115, 106601. 10.1016/j.foodhyd.2021.106601

[B12] Clemenson S. Muggeridge M. Clay M. (2012). “Quality specifications for herbs and spices,” in Handbook of herbs and spices. Second Edition, 25–41. 10.1533/9780857095671.25

[B13] Dai Y. Van Spronsen J. Witkamp G. J. Verpoorte R. Choi Y. H. (2013). Natural deep eutectic solvents as new potential media for green technology. Anal. Chim. Acta 766, 61–68. 10.1016/j.aca.2012.12.019 23427801

[B14] Davitadze N. (2023). Modification of the process of obtaining pectin by the methods of membrane technology. J. Ecol. Eng. 24 (11), 117–126. 10.12911/22998993/171469

[B15] Denman L. J. Morris G. A. (2015). An experimental design approach to the chemical characterisation of pectin polysaccharides extracted from *Cucumis melo* Inodorus. Carbohydr. Polym. 117, 364–369. 10.1016/j.carbpol.2014.09.081 25498647

[B16] Ding H. Cui S. W. (2020). Pectin bioactivity. Pectin Technol. Physiological Prop., 165–188. 10.1007/978-3-030-53421-9_9

[B17] Dranca F. Vargas M. Oroian M. (2020). Physicochemical properties of pectin from *Malus domestica* ‘Fălticeni’ apple pomace as affected by non-conventional extraction techniques. Food Hydrocoll. 100, 105383. 10.1016/j.foodhyd.2019.105383

[B18] Dubois M. Gilles K. A. Hamilton J. K. Robers P. A. Smith F. (1956). Colorimetric method for determination of sugars and related substances. Anal. Chem. 28, 350–356. 10.1021/ac60111a017

[B19] Einhorn-Stoll U. Kastner H. Hecht T. Zimathies A. Drusch S. (2015). Modification and physico-chemical properties of citrus pectin – influence of enzymatic and acidic demethoxylation. Food Hydrocoll. 51, 338–345. 10.1016/j.foodhyd.2015.05.031

[B20] Einhorn-Stoll U. Kunzek H. (2009). Thermoanalytical characterisation of processing-dependent structural changes and state transitions of citrus pectin. Food Hydrocoll. 23 (1), 40–52. 10.1016/j.foodhyd.2007.11.009

[B21] Einhorn-Stoll U. Kunzek H. Dongowski G. (2007). Thermal analysis of chemically and mechanically modified pectins. Food Hydrocoll. 21, 1101–1112. 10.1016/j.foodhyd.2006.08.004

[B22] Escudero Álvarez E. González Sánchez P. (2006). Dietary fibre. Nutr. Hosp. 21 (2), 61–72. 16771074

[B23] FAO (2012). Pérdidas y desperdicio de alimentos en el mundo – Alcance, causas y prevención. Available at: https://www.fao.org/4/i2697s/i2697s.pdf (Accessed June 25, 2024).

[B24] Filisetti-Cozzi T. M. C. C. Carpita N. C. (1991). Measurement of uronic acids without interference from neutral sugars. Anal. Biochem. 197, 157–162. 10.1016/0003-2697(91)90372-z 1952059

[B25] Fissore E. N. Matkovic L. Wider E. Rojas A. M. Gerschenson L. N. (2009). Rheological properties of pectin-enriched products isolated from butternut (*Cucurbita moschata Duch* ex *Poiret*). LWT - Food Sci. Technol. 42 (8), 1413–1421. 10.1016/j.lwt.2009.03.003

[B26] Food and Agriculture Organization of the United Nations (2024). FAOSTAT database. Rome, Italy: FAO. Available at: https://www.fao.org/faostat/en/#data/QCL (Accessed September 2, 2024).

[B27] Gawkowska D. Cybulska J. Zdunek A. (2018). Structure-related gelling of pectins and linking with other natural compounds: a review. Polymers 10 (7), 762. 10.3390/polym10070762 30960687 PMC6404037

[B28] George N. Andersson A. A. M. Andersson R. Kamal-Eldin A. (2020). Lignin is the main determinant of total dietary fiber differences between date fruit (*Phoenix dactylifera* L.) varieties. NFS J. 21, 16–21. 10.1016/j.nfs.2020.08.002

[B29] Gerschenson L. N. Fissore E. N. Rojas A. M. Idrovo Encalada A. M. Zukowski E. F. Higuera Coelho R. A. (2021). Pectins obtained by ultrasound from agroindustrial by-products. Food Hydrocoll. 118 (106799), 106799. 10.1016/j.foodhyd.2021.106799

[B30] Gómez Vargas C. B. Otálora González C. Gerschenson L. N. (2023). Reología de solventes eutécticos naturales para el aislamiento de bioactivos. XVIII Congr. Argent. De. Cienc. Y Tecnol. De. Aliment. XVIII CyTAL®, 2023.

[B31] Hammi K. M. Hammami M. Rihouey C. Le Cerf D. Ksouri R. Majdoub H. (2018). GC-EI-MS identification data of neutral sugars of polysaccharides extracted from Zizyphus lotus fruit. Data Brief 18, 680–683. 10.1016/j.dib.2018.01.085 29896535 PMC5995744

[B32] Hansen B. B. Spittle S. Chen B. Poe D. Zhang Y. Klein J. (2021). Deep eutectic solvents: a review of fundamentals and applications. Chem. Rev. 121 (3), 1232–1285. 10.1021/acs.chemrev.0c00385 33315380

[B33] Heredia-Guerrero J. A. Benítez J. J. Dominguez E. Bayer I. S. Cingolani R. Athanassiou A. (2014). Infrared and Raman spectroscopic features of plant cuticles: a review. Front. Plant Sci. 5, 305. 10.3389/fpls.2014.00305 25009549 PMC4069575

[B34] Houben K. Jolie R. P. Fraeye I. Van Loey A. M. Hendrickx M. E. (2011). Comparative study of the cell wall composition of broccoli, carrot, and tomato: structural characterization of the extractable pectins and hemicelluloses. Carbohydr. Res. 346, 1105–1111. 10.1016/j.carres.2011.04.014 21536260

[B35] Hu W. Ye X. Chantapakul T. Chen S. Zheng J. (2020). Manosonication extraction of RG-I pectic polysaccharides from citrus waste: optimization and kinetics analysis. Carbohydr. Polym. 235, 115982. 10.1016/j.carbpol.2020.115982 32122512

[B36] Idrovo Encalada A. Pérez C. Flores S. Rossetti L. Fissore E. Rojas A. M. (2019). Antioxidant pectin enriched fractions obtained from discarded carrots (Daucus carota L.) by ultrasound-enzyme assisted extraction. Food Chem. 289, 453–460. 10.1016/j.foodchem.2019.03.078 30955636

[B38] International Pectin Producers Association (IPPA) (2024). The molecular structure of pectin. Available at: https://pectinproducers.com/[Accessed [Accessed May 2024]

[B39] Koziol A. Sroda-Pomianek K. Górniak A. Wikiera A. Cyprych K. Malik M. (2022). Structural determination of pectins by spectroscopy methods. Coatings 12 (4), 546. 10.3390/coatings12040546

[B40] Lowry O. H. Rosebrough N. J. Farr A. L. Randall R. J. (1951). Protein measurement with the Folin phenol reagent. J. Biol. Chem. 193, 265–275. 10.1016/s0021-9258(19)52451-6 14907713

[B41] Luo J. Xu Y. Fan Y. (2019). Upgrading pectin production from apple pomace by acetic acid extraction. Appl. Biochem. Biotechnol. 187 (4), 1300–1311. 10.1007/s12010-018-2893-1 30218302

[B42] Marcon M. V. Vriesmann L. C. Wosiacki G. Beleski-Carneiro E. Petkowicz C. L. O. (2005). Pectins from apple pomace. Polímeros Ciência Tecnol. 15 (2), 127–129. 10.1590/s0104-14282005000200012

[B43] Morales-Contreras B. E. Wicker L. Rosas-Flores W. Contreras-Esquivel J. C. Gallegos-Infante J. A. Reyes-Jaquez D. (2020). Apple pomace from variety “Blanca de Asturias” as sustainable source of pectin: Composition, rheological, and thermal properties. LWT 117, 108641. 10.1016/j.lwt.2019.108641

[B44] Morris V. J. Belshaw N. J. Waldron K. W. Maxwell E. G. (2013). The bioactivity of modified pectin fragments. Bioact. Carbohydrates Diet. Fibre 1 (1), 21–37. 10.1016/j.bcdf.2013.02.001

[B45] Ng A. Parr A. J. Ingham L. M. Rigby N. M. Waldron K. W. (1998). Cell wall chemistry of carrots (Daucus carota Cv. Amstrong) during maturation and storage. J. Agric. Food Chem. 46, 2933–2939. 10.1021/jf9709921

[B46] Niu H. Dou Z. Hou K. Wang W. Chen X. Chen X. (2023). A critical review of RG-I pectin: sources, extraction methods, structure, and applications. Crit. Rev. Food Sci. Nutr. 64 (24), 8911–8931. 10.1080/10408398.2023.2204509 37114929

[B47] Otálora C. M. Bonifazi E. L. Fissore E. N. Basanta M. F. Gerschenson L. N. (2020). Thermal stability of betalains in by-products of the blanching and cutting of *Beta vulgaris* L. var *conditiva*. 2020. Pol. J. Food Nutr. Sci. 70 (1), 15–24. 10.31883/pjfns/116415

[B48] Ravindran R. Hassan S. S. Williams G. A. Jaiswal A. K. (2018). A review on bioconversion of agro-industrial wastes to industrially important enzymes. Bioengineering 5 (4), 93. 10.3390/bioengineering5040093 30373279 PMC6316327

[B49] Riyamol G. Jeevitha G. C. (2024). Microwave and ultrasound-assisted natural deep eutectic solvents-based extraction of pectin from onion peel wastes. CyTA - J. Food 22 (1). 10.1080/19476337.2024.2311215

[B50] Ruano P. Lazo Delgado L. Picco S. Villegas L. Tonelli F. Aguilera Merlo M. E. (2020). Extraction and characterization of pectins from peels of criolla oranges (*Citrus sinensis*). Exp. Rev. 10.5772/intechopen.88944

[B51] Salato G. Ponce N. M. A. Raffo D. Vicente A. R. Storz C. A. (2013). Developmental changes in cell wall polysaccharides from sweet cherry (Prunus avium L.) cultivars with contrasting firmness. Postharvest Biol. Technol. 84, 66–73. 10.1016/j.postharvbio.2013.04.009

[B52] Santana-Mayor A. Rodríguez-Ramos R. Herrera-Herrera A. V. Socas-Rodríguez B. Rodríguez-Delgado M. A. (2021). Deep eutectic solvents. The new generation of green solvents in analytical chemistry. Trends Anal. Chem. 134, 116108. 10.1016/j.trac.2020.116108

[B53] Santo Domingo C. Soria M. Rojas A. M. Fissore E. N. Gerschenson L. N. (2015). Protease and hemicellulase assisted extraction of dietary fiber from wastes of *Cynara cardunculus. International Journal of Molecular Sciences* . Int. J. Mol. Sci. 16 (3), 6057–6075. 10.3390/ijms16036057 25809605 PMC4394519

[B67] Sokal R. R. Rohlf J. B. (2000). Biometry. The Principles and Practice of Statistics in Biological Research. San Francisco: WH Freeman and Company.

[B66] Tien N. N. T. Le N. L. Khoi K. T. Richel A. (2022). Characterisation of dragon fruit peel pectin extracted with natural deep eutectic solvent and sequential microwave-ultrasound-assisted approach. IJFST 57 (6), 3735–3749. 10.1111/ijfs.15699

[B55] Wang W. Ma X. Jiang P. Hu L. Zhi Z. Chen J. (2016). Characterization of pectin from grapefruit peel: a comparison of ultrasound-assisted and conventional heating extractions. Food Hydrocoll. 61, 730–739. 10.1016/j.foodhyd.2016.06.019

[B56] Wang X. Chen Q. Lü X. (2014). Pectin extracted from apple pomace and citrus peel by subcritical water. Food Hydrocoll. 38, 129–137. 10.1016/j.foodhyd.2013.12.003

[B57] Wani K. M. Uppaluri R. V. S. (2023). Characterization of pectin extracted from pomelo peel using pulsed ultrasound assisted extraction and acidic hot water extraction process. Appl. Food Res. 3 (7), 100345. 10.1016/j.afres.2023.100345

[B58] Wikiera A. Kozioł A. Mika M. Stodolak B. (2022). Structure and bioactivity of apple pectin isolated with arabinanase and mannanase. Food Chem. 388, 133020. 10.1016/j.foodchem.2022.133020 35483285

[B60] Wu Y. C. Wu P. Li Y. B. Liu T. C. Zhang L. Zhou Y. H. (2018). Natural deep eutectic solvents as new green solvents to extract anthraquinones from Rheum palmatum L. RSC Adv. 8 (27), 15069–15077. 10.1039/C7RA13581E 35541349 PMC9079993

[B61] Xu F. Zhang S. Waterhouse G. I. N. Zhou T. Du Y. Sun-Waterhouse D. (2022). Yeast fermentation of apple and grape pomaces affects subsequent aqueous pectin extraction: composition, structure, functional and antioxidant properties of pectins. Food Hydrocoll. 133, 107945. 10.1016/j.foodhyd.2022.107945

[B62] Zhao X. Zhou Y. Liu J. Chen J. Ye F. Zhao G. (2020). Effects of sucrose on the structure formation in high-methoxyl apple pectin systems without acidifier. Food Hydrocoll. 105, 105783. 10.1016/j.foodhyd.2020.105783

[B63] Zheng J. Li H. Wang D. Li R. Wang S. Ling B. (2021). Radio frequency assisted extraction of pectin from apple pomace: process optimization and comparison with microwave and conventional methods. Food Hydrocoll. 121, 107031. 10.1016/j.foodhyd.2021.107031

[B64] Zhou J. Liu D. Xia W. Guo Y. Luo Y. Xue J. (2023). Physicochemical and functional properties of RG-I enriched pectin extracted from thinned-young apples. Int. J. Biol. Macromol. 236, 123953. 10.1016/j.ijbiomac.2023.123953 36898465

[B65] Zlatanović S. Ostojić S. Micić D. Rankov S. Dodevska M. Vukosavljević P. (2019). Thermal behaviour and degradation kinetics of apple pomace flours. Thermochim. Acta 673, 17–25. 10.1016/j.tca.2019.01.009

